# Sputum Biomarkers of Inflammation to Track Acute Respiratory Events in School-Age Children with Cystic Fibrosis

**DOI:** 10.3390/ijms27031616

**Published:** 2026-02-06

**Authors:** Elad Ben-Meir, Lucy Perrem, Gyde Nissen, Michelle Shaw, Felix Ratjen, Hartmut Grasemann

**Affiliations:** 1 Pediatric Pulmonary Unit, Hadassah Medical Center, Faculty of Medicine, Hebrew University of Jerusalem, Jerusalem 91120, Israel; eladbm@hadassah.org.il; 2 Division of Respiratory Medicine, Children’s Health Ireland, University College Dublin, D01 YC67 Dublin, Ireland; 3 Department of Paediatrics, University Hospital Schleswig-Holstein, University of Lübeck, 23562 Lübeck, Germany; 4 Division of Respiratory Medicine, Department of Paediatrics, The Hospital for Sick Children, Toronto, ON M5G 1E8, Canada; 5 Translational Medicine, Research Institute, The Hospital for Sick Children, University of Toronto, Toronto, ON M5G 1E8, Canada

**Keywords:** cystic fibrosis, acute respiratory events, airway inflammation, neutrophilic inflammation, cytokines, calprotectin, IL-1 pathway, biomarkers

## Abstract

Cystic fibrosis (CF) is characterized by neutrophil-driven airway inflammation and acute respiratory events (AREs) that contribute to progressive lung damage. AREs are clinically heterogeneous and often occur without measurable changes in lung function. This study aimed to evaluate the utility of molecular airway inflammatory markers for detecting AREs in school-age children with CF. We performed a secondary analysis of a prospective observational study of children with CF (ages 6.7–16.8 years) followed for two years. Sputum samples were collected from 50 participants during stable visits and AREs. Concentrations of 14 inflammatory cytokines were measured using ELISA and multiplex assays. Associations with lung function (ppFEV_1_ and lung clearance index [LCI]) and time to next ARE were assessed. A total of 179 sputum samples were analyzed, including 64 collected during AREs. Calprotectin, interleukin-8 (IL-8), and IL-1β were increased during AREs compared with stable visits, although concentrations frequently remained within ranges observed at stable visits. Other cytokines, including GM-CSF, IL-17A, IL-1α, TNF-α, and SPLUNC-1, were predictive of shorter time to subsequent AREs. No biomarker correlated with lung function measures. These findings indicate that airway inflammatory cytokine changes are associated with clinically diagnosed AREs but not with pulmonary function, supporting their potential role as complementary biomarkers in CF care.

## 1. Introduction

Cystic fibrosis (CF) lung disease is characterized by inflammation, as well as acute and chronic bacterial infections of the airways, leading to mucus plugging, bronchiectasis and progressive loss of pulmonary function [1]. Pulmonary exacerbations, which are typically caused by infections, contribute importantly to disease progression [2,3,4,5]. In children with CF, such respiratory events range widely in severity and clinical presentation [6,7,8], and distinguishing self-resolving viral infections from lower respiratory tract events requiring antibiotic therapy is challenging. As a result, clinicians often adopt a low threshold for prescribing antibiotics, leading not only to potential overuse but also substantial variability in management strategies across providers and CF centers [9].

Spirometry is still the most commonly used assessment tool to guide treatment decisions in school-age children with CF, as an acute decline in forced expiratory volume in one second (FEV_1_), in combination with clinical symptoms, usually prompts the initiation of antibiotic therapy. However, spirometry lacks sensitivity in early CF lung disease and most acute respiratory events (AREs) in CF children are not associated with significant spirometric changes [6]. The lung clearance index (LCI), derived from the multiple breath washout (MBW) test and now an established outcome measure in interventional trials, offers greater sensitivity than FEV_1_ in early CF lung disease. We recently demonstrated that the LCI worsens during AREs in preschool-aged children with CF, improves with antibiotic treatment [10], and can detect incomplete recovery at follow-up [11]. However, availability of MBW is limited in routine clinical settings. Consequently, there is a need for additional, sensitive biomarkers to detect AREs, a clinical need that may be amplified in the era of effective CFTR modulators.

Neutrophil-predominant airway inflammation is central to CF lung disease progression, and numerous cytokines have been implicated as biomarkers of disease [12,13,14,15], with neutrophil elastase (NE) and IL-8 among the best established. Significantly elevated NE activity is observed early in life and contributes to airway injury and structural damage. In pediatric CF cohorts, increased NE correlates with early lung injury on imaging [16], and sputum NE activity correlates closely with lung disease severity [17]. NE, together with matrix metalloproteinase-12 released from activated neutrophils and macrophages, plays a key role in initiating and perpetuating CF airway pathology [18]. In infants and young children with CF, NE in bronchoalveolar lavage fluid correlates with bronchiectasis formation on CT imaging [19,20]; longitudinal studies show that early and persistent NE elevation predicts the progression of structural lung disease more strongly than infection burden [21], and sputum NE inversely associates with FEV_1_ in older children [22]. The role of airway NE in detecting new-onset AREs, however, is not clear [23].

Other markers of neutrophilic inflammation, including tumor necrosis factor-α (TNF-α) [24], interleukin-1α (IL-1α) and IL-1β [15,16], IL-6, IL-8, IL-17A [22,24,25], granulocyte colony-stimulating factor (G-CSF) [26], granulocyte–macrophage colony-stimulating factor (GM-CSF) [26,27], and calprotectin [28,29], are also elevated in CF airways, while counter-regulatory mediators such as IL-10 are often deficient [30]. Although these inflammatory mediators have been investigated as potential biomarkers of CF lung disease activity, their relationship to clinical symptoms and established physiological measures remains poorly defined.

We therefore aimed to evaluate the relation of specific inflammatory cytokine concentrations (i.e., the IL-1 pathway-related IL-1α, IL-1β, IL-1RA, IL-6, IL-8, and IL-17A, as well as NE, calprotectin, IL-10, TNF-α, IFN-γ, G-CSF, GM-CSF, and SPLUNC1) in the sputum of school-aged CF children with clinically defined AREs and physiological measures of pulmonary function (FEV_1_ and LCI) during stable and symptomatic outpatient visits.

## 2. Results

A total of 179 sputum samples, which included 115 samples from stable visits and 64 samples from AREs, from 50 participants with CF were included. The participants’ demographic characteristics at enrollment are shown in Table 1. The mean age of the study population at enrollment was 12.2 years (range, 6.7–16.8 years) and 32% were males. Participants were followed for a median of 2 years (interquartile range [IQR], 1.9–2.0) and sputum samples were obtained from four (IQR, 3–5) visits per participant.

**Figure 1:**
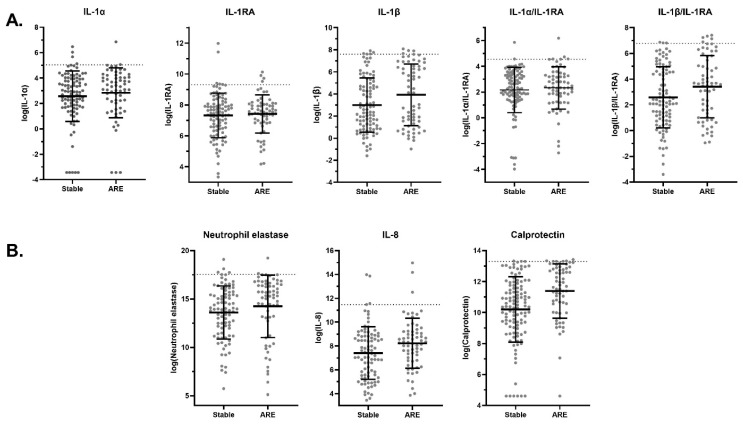
Inflammatory cytokine concentrations at stable visits and during acute respiratory events (AREs). (**A**) Interleukin-1 (IL-1) family cytokines and ratios, including IL-1α, IL-1β, IL-1 receptor antagonist (IL-1RA), IL-1α/IL-1RA, and IL-1β/IL-1RA. (**B**) Neutrophil-associated inflammatory markers, including neutrophil elastase (NE), interleukin-8 (IL-8), and calprotectin. Concentrations are presented on a natural logarithmic scale. Horizontal black lines denote the median with interquartile range (IQR). The dotted line across the *y*-axis represents the upper limit of normal (ULN) as calculated from the stable visits.

Several cytokine concentrations were elevated at AREs compared to stable visits (Figure 1, Table 2). Figure 1 highlights a selected subset of cytokines demonstrating the most pronounced differences between stable visits and acute respiratory events, whereas Table 2 provides a comprehensive overview of all analyzed biomarkers, including within-person changes and the proportion of ARE samples exceeding the upper limits of normal. Calprotectin demonstrated the most significant increase, with a mean difference of 1.00 log [pg/mL] (95% CI: 0.47 to 1.53, *p* < 0.001) between AREs and stable visits, and mean within-subject change of 1.21 (95% CI: 0.67 to 1.76, *p* < 0.001) at AREs (Figure 1B). Mean concentrations of IL-1β, TNFα, IFNγ, IL-10, IL-8, and the IL-1β/IL-RA ratio were also increased at AREs (*p* < 0.05) (Figure 1A). In contrast, G-CSF, GM-CSF, IL-17A, and SPLUNC-1 concentrations did not change with AREs. Of all cytokines quantified, concentrations in ARE samples exceeded the upper limit of normal (ULN) observed at stable visits in less than 10% of samples (Table 2).

**Figure 2:**
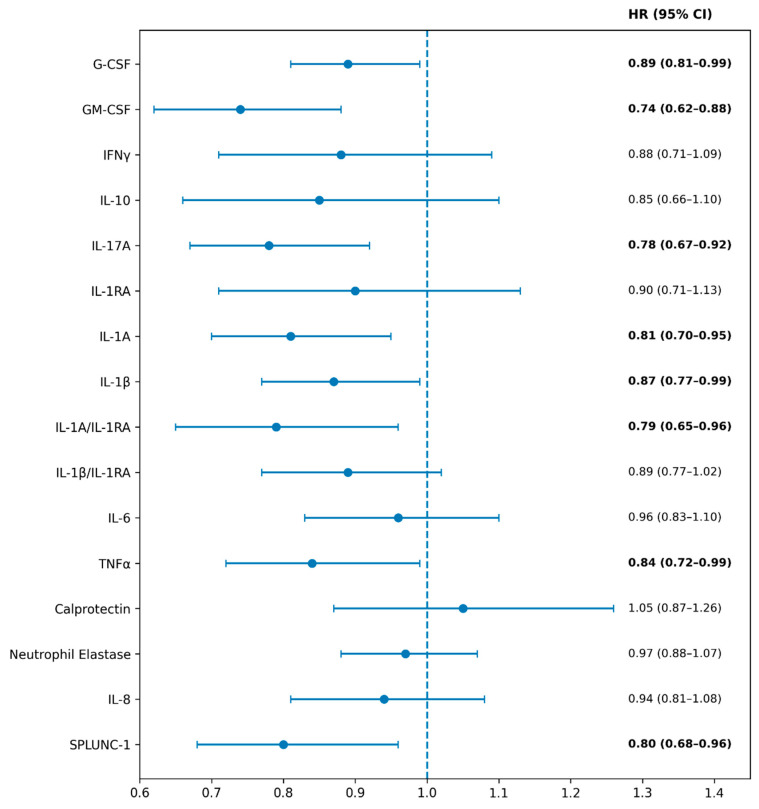
Biomarker prediction of acute respiratory events (AREs). Forest plot showing hazard ratios (HRs) and 95% confidence intervals (CIs) for time to next ARE associated with sputum inflammatory cytokine concentrations. HRs were estimated using univariable Cox proportional hazards models, with HR < 1 indicating a shorter time to ARE. Bold values denote statistically significant hazard ratios (95% confidence intervals excluding 1.0).

Several cytokines were associated with the risk of future AREs. GM-CSF, IL-17A, IL-1α, TNF-α, and SPLUNC-1 were all associated with shorter time to the next ARE, with hazard ratios ranging from 0.74 to 0.84 (*p* < 0.05) (Figure 2). No correlations were found between sputum biomarker levels and lung function measurements (Table 3).

## 3. Discussion

In this study, we evaluated sputum cytokine concentrations as potential airway inflammatory biomarkers in school-age children with cystic fibrosis during stable clinical states and acute respiratory events (AREs). Several cytokines had higher mean concentrations at AREs compared to stable visits, and significant within-person increases from the previous stable visit to ARE. Calprotectin showed the largest mean concentration difference and the most significant increase from stable to ARE. However, despite the observed differences and increases in mean concentrations during AREs of calprotectin, IL-8, and IL-1β, substantial overlap with concentrations observed at stable visits limits their discriminatory utility when interpreted as absolute values. Indeed, fewer than 10% of ARE samples exceeded upper limits of concentration derived from stable visits. These findings indicate that single biomarker thresholds are unlikely to support real-time diagnostic decision-making at the individual patient level. Instead, our data support a longitudinal interpretation framework, in which within-person changes relative to a prior stable baseline, or population-level risk stratification, may provide greater clinical value. Importantly, these findings arise from an observational study design and should be interpreted as reflecting associations rather than defining underlying mechanistic pathways.

Calprotectin is one of the most abundant cytosolic proteins in neutrophils and is released during neutrophil activation, degranulation, cell death, and neutrophil extracellular trap formation [31,32,33]. Acting as both a damage-associated molecule and antimicrobial mediator through metal chelation, calprotectin plays an active role in innate immune signaling rather than serving solely as a passive marker of inflammation [31,32,33,34]. In CF, calprotectin closely reflects neutrophilic inflammatory burden and typically parallels other neutrophil-associated biomarkers, such as IL-8 and NE, while capturing complementary aspects of neutrophil activation rather than protease concentration or activity alone [33,34,35]. Previous studies have demonstrated elevated calprotectin levels in CF airway secretions and blood, with sputum calprotectin decreasing following treatment of pulmonary exacerbations, and serum calprotectin showing potential value in predicting time to subsequent exacerbation [28,29,36,37]. Together with our findings of marked calprotectin elevation during AREs in children with CF, these data support calprotectin as a biologically plausible and clinically relevant biomarker of acute CF airway inflammation.

Other neutrophil-associated mediators, including NE, IL-8, IL-6, IL-17A, IL-10, and TNF-α, have also been identified previously to contribute to CF airway inflammation. NE is a major driver of airway damage, correlating strongly with lung function decline and early structural disease [16,17,18,19,20,22]. IL-8 is the dominant neutrophil chemoattractant and is closely associated with airway obstruction [15,21,22,27], while IL-6 reflects ongoing epithelial and immune activation [15,19,20,25]. IL-17A, a Th17 cytokine, promotes sustained neutrophil recruitment and has been linked to infection burden and structural progression [20,38]. IL-10 provides counter-regulation but is often insufficient in CF airways [24,30], while TNF-α amplifies neutrophil-directed inflammation [39]. Together, these mediators form a tightly interconnected network that perpetuates chronic inflammation. Our results support this framework and show that in addition to calprotectin, TNF-α, IL-6, IL-8 and IL-10 are also increased at AREs compared to stable visits, suggesting that fluctuations in these cytokines may reflect biological sensitivity to events where physiological pulmonary function test results (FEV_1_ and LCI) remain unchanged.

Another group of cytokines that have previously been identified as central drivers of neutrophilic airway inflammation in CF, are the IL-1 pathway-related cytokines (IL-1α, IL-1β, IL-1RA, IL-6, IL-8, and IL-17A) [19,20,21,22,38,40,41,42,43,44,45,46,47,48]. IL-1β is consistently reported to be elevated in CF sputum and is rapidly released after epithelial injury or bacterial stimulation, inducing downstream mediators through NF-κB activation [40]. Dysregulated NLRP3 inflammasome activity in CFTR-deficient cells promotes excessive IL-1β secretion [41,42], while IL-1α functions as an epithelial alarmin that amplifies early neutrophil recruitment [43]. IL-1β also increases mucin production and goblet cell hyperplasia, further impairing mucociliary clearance. We recently demonstrated that IL-1β reductions following therapy with ivacaftor correlated with improvements in airway nitric oxide (NO) and lung function, linking IL-1 pathway-driven inflammation to CF-related molecular NO deficiency [44]. Our current findings contribute to the existing body of work by showing that IL-1 pathway-related cytokines not only change with AREs but may also provide prognostic information. In our cohort, IL-1β was increased during AREs, while higher IL-1α, and to a lesser extent IL-1β levels at stable visits, predicted shorter time to subsequent AREs. These signal changes were unrelated to changes in measures of lung function (spirometry and LCI), suggesting that IL-1-mediated inflammation could therefore potentially be used as a biomarker to identify AREs before physiological changes become apparent.

Although not consistently increased during AREs, higher GM-CSF levels at stable visits predicted shorter time to the next ARE, suggesting that persistent activation of myeloid pathways reflects underlying airway vulnerability. GM-CSF and G-CSF are regulated to neutrophil maturation and survival and are elevated in CF airways in response to bacterial and epithelial stimuli [26]. Interferon-γ (IFN-γ), a key Th1 cytokine, is generally reduced in CF sputum and BAL fluid, reflecting a blunted Th1 response in a disease dominated by innate neutrophilic inflammation [14,25,49]. A recent study reported that higher baseline IFN-γ was unexpectedly associated with better early improvement in lung function during treatment for CF PEx, suggesting that preserved Th1 responsiveness may contribute to treatment responsiveness [49]. In our cohort, IFN-γ showed only modest, inconsistent ARE-related changes and no meaningful association with lung function. 

SPLUNC1 is an epithelial defense protein that regulates airway surface liquid homeostasis, inhibits ENaC, and contributes to mucosal defense. SPLUNC1 levels are reduced in CF due to impaired epithelial secretion and proteolytic degradation, and low levels are associated with airway dehydration and exacerbation risk [50,51,52,53]. In a recently published pediatric study, it was shown that SPLUNC1 was decreased during pulmonary exacerbation (PEx) episodes, increased with antibiotic therapy, and that higher stable visit levels predicted longer PEx-free intervals [54]. In the present cohort, SPLUNC1 did not differ between AREs and stable visits, suggesting that SPLUNC1, while potentially informative for detection and monitoring of CF PEx, is insensitive to detect AREs. However, lower SPLUNC1 at stable visits predicted shorter time to the next ARE, matching our previous observation for PEx, maybe indicating that reduced SPLUNC1 reflects impaired epithelial defense and increased susceptibility to symptomatic worsening. 

In addition to SPLUNC1 and GM-CSF, a limited number of additional cytokines also demonstrated meaningful value to predict AREs. Higher concentrations of IL-1α, IL-17A, and TNF-α at stable visits were all associated with shorter time to the next ARE, suggesting that activation of epithelial danger signaling and the Th17 pathway may precede clinical evidence of AREs. These findings underscore the potential value of inflammatory biomarkers in identifying clinical instability beyond physiological measurements such as FEV_1_ and LCI. Interestingly, calprotectin was not among the markers associated with time to next ARE.

The absence of correlations between sputum inflammatory markers and ppFEV_1_ or LCI may be explained by several factors. Spirometry is insensitive to early or acute inflammatory changes in pediatric CF, and while LCI is more sensitive than FEV_1_, such physiological measures may lag behind molecular inflammatory activity. Additionally, sputum sampling and lung function testing may not be temporally synchronized with peak inflammatory responses. Finally, heterogeneity in disease severity, airway involvement, and treatment exposure within this school-age cohort may further attenuate correlations. Together, these findings suggest that monitoring airway inflammation may provide complementary information to current clinical standards.

Our study has limitations. A key limitation is the increasing difficulty of obtaining expectorated sputum samples in children with CF in the era of CFTR modulator therapy. As many children treated with modulators have reduced sputum production, this limits the feasibility and generalizability of sputum-based biomarker assessment in contemporary pediatric CF populations. Consequently, while sputum biomarkers remain biologically informative, their clinical applicability may be reduced, underscoring the need for alternative airway sampling strategies. Moreover, our predominantly pre-modulator cohort may also have had higher baseline airway inflammation than is typical for CF patients treated with CFTR modulators today. Sputum samples were obtained at discrete clinical time points and therefore cannot fully capture the dynamic and heterogeneous nature of CF airway inflammation. Moreover, expectorated sputum may not adequately represent distal airway disease, or focal parenchymal processes. As such, sputum bio-markers should be interpreted as surrogate measures of airway inflammation and considered complementary to physiological testing and imaging-based assessments. The modest sample size, reliance on spontaneous sputum expectoration, and exploratory nature of multiple comparisons may limit the generalizability of our results. In addition, our definition of the upper limits of normal ranges (ULNs) may be conservative, reflecting potential selection bias toward higher baseline levels of inflammation in CF children able to expectorate sputum.

Important clinical variables, including airway infection status, antibiotic exposures, corticosteroid use, and CFTR modulator therapy, were not incorporated as covariates in the present analysis but may have affected inflammatory profiles and clinical outcomes. In particular, our predominantly pre-modulator cohort may exhibit higher baseline airway inflammation than contemporary CF populations treated with highly effective CFTR modulators. Importantly, this study did not include a healthy control group, limiting our ability to directly compare cytokine concentrations with non-CF baseline levels. Nevertheless, consistent patterns across markers and outcomes support the internal validity of our findings and provide a valuable reference for unmodulated inflammatory signatures in CF to inform future research and is of potential value for people with CF not currently treated with CFTR modulators.

## 4. Materials and Methods

### 4.1. Study Cohort Details

This was a secondary analysis of a previously published study which occurred from April 2017–February 2020 at The Hospital for Sick Children in Toronto, Canada and Riley Hospital for Children in Indianapolis, USA [11]. This parent study was a prospective observational trial in school-age CF children (5–16.8 years at enrolment). Participants were followed every 3 months for two years, in keeping with routine clinical follow-up, as well as during acute respiratory events, defined as any symptomatic visit, and were categorized into four visit types on the basis of treatment status [11]. In this analysis, we included two symptomatic visit types (“pulmonary exacerbation” and “increased cough event”) under the term acute respiratory event (ARE). Stable visits were defined as asymptomatic outpatient visits without clinical evidence of respiratory worsening. At each study visit, a clinical history and physical exam were obtained by a CF physician, and multiple breath washout (MBW) and spirometry were attempted. Airway infection status, antibiotic use, and corticosteroid exposure were managed according to standard clinical care and were not protocolized as part of this observational study. CFTR modulator therapy was documented at enrollment, with the majority of participants enrolled prior to widespread use of highly effective CFTR modulator therapy. All children who were followed at The Hospital for Sick Children, and were able to spontaneously expectorate sputum, were eligible for inclusion in the present analysis. No additional inclusion or exclusion criteria related to sputum collection were applied. Expectorated sputum samples were collected in sterile containers and subdivided for routine microbiology testing and for inflammatory cytokine measurements. The current study analyzed the airway markers of inflammation in subjects with at least one banked sputum sample (see below).

The present study extends the original investigation by comprehensively characterizing airway inflammatory cytokine profiles in bio-banked sputum samples and examining their associations with acute respiratory events and future event risk. These inflammatory analyses and outcome associations were not evaluated in the previously published report, which focused primarily on physiological changes in the lung clearance index (LCI) during acute respiratory events.

### 4.2. Sputum Processing and Biomarker Analysis

Spontaneously expectorated sputum samples were processed within two hours according to established protocols [21], and supernatants of homogenized mucous plugs were stored at −80 °C prior to analysis. While sputum samples were collected prospectively as part of the original observational study, inflammatory biomarker measurements were performed after completion of participant follow-up as part of this predefined secondary analysis. A total of 14 inflammatory proteins involved in neutrophil-driven CF airway inflammation were quantified in sputum supernatant. Milliplex kits were used for the quantification of IL-8 (HCYTOMAG-60K-01 Human Cytokine/Chemokine Magnetic Kit, Millipore Sigma, Burlington, MA, USA) and NE (HSP3MAG-63K-01, Human Sepsis Magnetic Panel 3, Millipore Sigma, Burlington, MA, USA), and a multiplex assay was used to measure levels of G-CSF, GM-CSF, IL-1α, IL-1ß, IL-1RA, IL-10, IL-6, IL-17α, IFN-y and TNF-α (HCYTOMAG-60K-10 Human Cytokine/Chemokine Magnetic Kit, Millipore Sigma, Burlington, MA, USA). Commercially available ELISA kits were used for the quantification of calprotectin (DS8900 Human S100A8/S100A9 Heterodimer ELISA Kit, R&D Systems, Minneapolis, MN, USA) and SPLUNC1 (RAB1173 Human PLUNC ELISA Kit, Sigma-Aldrich, St. Louis, MO, USA).

### 4.3. Lung Function

Pulmonary function was assessed by spirometry at each study visit in all three groups and the percent predicted forced expiratory volume in one second (ppFEV_1_) was calculated using published Global Lung Function Initiative (GLI) equations [55]. The nitrogen multiple breath washout (MBW) test was performed at visits in groups 1 and 3, using the Exhalyzer D (Ecomedics) device and the results were analyzed by Spiroware 3.3.2 software, according to the American Thoracic Society (ATS) and European Respiratory Society (ERS) consensus statement [56,57].

### 4.4. Statistical Analysis

Due to the skewed nature of the sputum cytokine measurements, values were transformed using natural logarithms. Values that were below the detectable limit were set to said limit, where applicable. Upper limits of normal ranges (ULNs) were defined using the sputum marker measurements at stable visits. We then assessed the proportion of ARE visits that fell above each marker’s ULN.

Analyses including differences between groups and relationships between changes in lung function and changes in sputum markers were assessed using linear regressions with generalized estimating equations to account for repeated measures within an individual. Time to next ARE was assessed using Cox proportional hazard models.

## 5. Conclusions

Distinct sputum biomarker changes from stable baseline reflect inflammatory activity during AREs and may help identify CF children at risk for future clinical instability. Calprotectin, IL-8, and IL-1β emerged as the most promising biomarkers for detecting AREs, whereas IL-1α, IL-17A, and GM-CSF demonstrated the strongest predictive value for identifying CF children at risk for future AREs. Because the inflammatory signals occurred independently of FEV_1_ and LCI changes, cytokine concentrations in CF airways secretions may provide important complementary insight beyond conventional physiological assessments and routine clinical assessments. Despite the growing challenge of sputum collection in the CFTR modulator era, our findings offer value beyond hypothesis generation and support further prospective studies to determine which airway inflammation biomarkers are sensitive and applicable measures to detect and monitor acute events in early CF lung disease.

## Figures and Tables

**Table 1:** Demographics of study population at enrollment.

	Participants (N = 50)
**Age at enrollment, years, mean (range)**	12.2 (6.7–16.8)
**Male, *n* (%)**	16 (32)
**Genotype functional class, *n* (%)**	
Class I–III	47 (94)
Class IV–V	3 (6)
LCI, units, mean (SD) *	9.4 (2.6)
ppFEV_1_, mean (SD) *	93.3 (16.1)
BMI centile, mean (SD) *	41.1 (21.9)
**Medication use at enrollment, *n* (%)**	
Hypertonic saline	27 (54)
Dornase alfa	29 (58)
Azithromycin	9 (18)
CFTR modulator	17 (34)

* Values taken from the first stable visit of the study.

**Table 2:** Differences and within-person changes in cytokine concentrations between stable visits and acute respiratory events (AREs). Differences represent mean differences in log-transformed biomarker concentrations between all stable visits and all AREs, with corresponding 95% confidence intervals (CIs). Changes represent within-person mean changes from the most recent stable visit to a subsequent ARE, with 95% CIs. Values are presented on a natural logarithmic scale. The proportion of ARE samples with biomarker concentrations exceeding the upper limit of normal (ULN), defined from stable visits, is shown.

Biomarker	Difference Between Stable and ARE			Change from Stable to ARE			Proportion of AREs with Biomarker Levels Above ULN
	Log [pg/mL]	95% CI	*p*-Value	Log [pg/mL]	95% CI	*p*-Value	*n* (%)
**G-CSF**	0.68	−0.23 to 1.58	0.14	**1.74**	**0.88 to 2.60**	**<0.001**	4/64 (6)
**GM-CSF**	0.09	−0.47 to 0.65	0.75	0.32	−0.29 to 0.93	0.30	4/64 (6)
**IFNγ**	**0.57**	**0.03 to 1.10**	**0.04**	**0.57**	**0.00 to 1.15**	**0.05**	3/64 (5)
**IL-10**	**0.58**	**0.07 to 1.08**	**0.02**	**0.85**	**0.33 to 1.38**	**0.002**	6/64 (9)
**IL-17A**	0.23	−0.40 to 0.86	0.47	0.56	−0.09 to 1.22	0.09	4/64 (6)
**IL-1RA**	0.04	−0.38 to 0.46	0.84	0.53	−0.09 to 1.15	0.09	4/64 (6)
**IL-1α**	0.28	−0.29 to 0.86	0.34	**1.17**	**0.31 to 2.03**	**0.009**	2/64 (3)
**IL-1β**	**0.85**	**0.05 to 1.64**	**0.04**	**1.47**	**0.51 to 2.44**	**0.004**	4/64 (6)
**IL-1α/IL-1RA**	0.30	−0.20 to 0.80	0.23	−1.03	−6.27 to 4.20	0.24	5/64 (8)
**IL-1β/IL-1RA**	**0.81**	**0.09 to 1.52**	**0.03**	**0.94**	**0.14 to 1.74**	**0.02**	5/64 (8)
**IL-6**	0.46	−0.12 to 1.04	0.12	**0.93**	**0.27 to 1.59**	**0.007**	4/64 (6)
**TNFα**	**0.67**	**0.05 to 1.29**	**0.03**	**1.28**	**0.57 to 1.99**	**<0.001**	4/64 (6)
**Calprotectin**	**1.00**	**0.47 to 1.53**	**<0.001**	**1.21**	**0.67 to 1.76**	**<0.001**	5/64 (8)
**Neutrophil Elastase**	0.58	−0.31 to 1.48	0.20	0.62	−0.50 to 1.73	0.27	2/64 (3)
**IL-8**	**0.74**	**0.08 to 1.40**	**0.02**	**1.02**	**0.40 to 1.63**	**0.002**	3/64 (5)
**SPLUNC-1**	−0.15	−0.62 to 0.32	0.53	−0.15	−0.81 to 0.50	0.65	2/62 (3)

**Table 3:** Correlations (R) between log-transformed sputum biomarker concentrations and pulmonary function measures (percent predicted FEV_1_ [ppFEV_1_] or lung clearance index [LCI]).

Biomarker	Correlation Between Cytokines and ppFEV1		Correlation Between Cytokines and LCI	
	R	*p*-Value	R	*p*-Value
**G-CSF**	−0.08	0.65	−0.01	0.96
**GM-CSF**	−0.29	0.07	0.25	0.12
**IFNγ**	0.03	0.84	0.16	0.31
**IL-10**	−0.09	0.59	0.12	0.48
**IL-17A**	−0.18	0.26	0.17	0.29
**IL-1RA**	0.07	0.65	−0.20	0.21
**IL-1α**	−0.07	0.69	0.03	0.85
**IL-1β**	−0.04	0.83	−0.19	0.24
**IL-1α/IL-1RA**	−0.14	0.41	0.20	0.21
**IL-1β/IL-1RA**	−0.09	0.58	−0.06	0.72
**IL-6**	0.09	0.61	−0.27	0.09
**TNFα**	0.02	0.92	−0.09	0.58
**Calprotectin**	−0.09	0.57	0.10	0.51
**Neutrophil Elastase**	−0.03	0.87	0.07	0.70
**IL-8**	0.19	0.27	−0.10	0.55
**SPLUNC-1**	0.08	0.66	0.06	0.71

## Data Availability

The original contributions presented in this study are included in the article. Further inquiries can be directed to the corresponding author.

## Abbreviations

The following abbreviations are used in this manuscript:

ARE	Acute respiratory event
ULN	Upper limit of normal
BMI	Body mass index
CFTR	Cystic fibrosis transmembrane conductance regulator.
ppFEV1	Precent predicted forced expiratory volume in first second
LCI	Lung clearance index
